# Tissue-Specific Lipid Profiles and Valorization Potential of Oils from Processing By-Products of the Invasive Blackchin Tilapia (*Sarotherodon melanotheron*)

**DOI:** 10.3390/foods15193444

**Published:** 2026-09-26

**Authors:** Thunnop Laokuldilok, Wannaporn Klangpetch, Sukhuntha Osiriphun, Nattapong Kanha, Kanyasiri Rakariyatham, Sukan Braspaiboon, Chalalai Jaisan

**Affiliations:** 1 Faculty of Agro-Industry, Chiang Mai University, Chiang Mai 50100, Thailand; 2 Cluster of High Value Products from Thai Rice and Plants for Health, Chiang Mai University, Chiang Mai 50200, Thailand; 3 Office of Research Administration, Chiang Mai University, Chiang Mai 50200, Thailand

**Keywords:** blackchin tilapia, fish oil, tissue-specific lipid profile, omega-3 fatty acids, volatile compounds

## Abstract

The global expansion of invasive aquatic species poses several ecological and economic challenges while generating underutilized biomass. Blackchin tilapia (*Sarotherodon melanotheron*) is an invasive fish species in Thailand, but its processing by-products remain poorly characterized as sources of tissue-specific lipids. This study evaluated the oil yield, fatty acid composition, nutritional quality indices (NQIs), lipid oxidation status, and volatile compound profiles of the oils recovered from the head, viscera, roe, and mixed processing by-products. The crude oils were characterized by gas chromatography–flame ionization detection and headspace gas chromatography–electronic nose analysis. The head produced the highest oil yield (17.5% dry-weight basis), whereas the roe oil showed relatively more favorable nutritional characteristics among the tissue-derived oils examined, with the highest relative polyunsaturated fatty acid level (14.8% of total fatty acids); the presence of eicosapentaenoic acid (EPA; 3.0% of total fatty acids) and docosahexaenoic acid (DHA; 7.4% of total fatty acids); a polyunsaturated fatty acid/saturated fatty acid ratio of 0.288; and a fish lipid quality index (10.47). The crude oils differed in their lipid oxidation status, with head oil showing the highest and roe oil the lowest oxidation indices. Volatile compound profiling resulted in the identification of 22 major compounds and revealed distinct tissue-dependent patterns, with 1-propanethiol as the predominant volatile compound in the roe oil (84.2%) and 2-hexanol as a major volatile compound in the head oil (23.6%). These findings demonstrate the distinct valorization potential of blackchin tilapia by-products and provide a basis for tissue-specific selection and further processing of invasive fish-derived oils.

## 1. Introduction

The spread of non-native fish species has become an increasingly serious ecological problem throughout the world, threatening biodiversity, aquaculture systems, and the livelihoods of local communities [1]. Among these species, the blackchin tilapia (*Sarotherodon melanotheron*), originally native to the brackish waters of West African lagoons, has successfully spread and established itself outside its natural distribution range in Asia, Europe, and North America [2]. This fish species is highly adaptable—able to tolerate wide variations in salinity and oxygen levels in the water—and often forms dense populations. Its omnivorous diet, along with its high reproductive capacity, further enhances the invasive potential of this fish species. Although primarily a brackish water fish, the blackchin tilapia can survive and reproduce in both freshwater and saltwater environments [3]. In Thailand, the rapid increase in the blackchin tilapia population has led to ecological, economic, and social problems, such as the decline of native fish populations, the degradation of water quality, and losses in the aquaculture sector [2]. By 2024, the invasive population had spread across more than 300 km^2^ in 13 provinces, with the population estimated to number in the millions [2].

Given that most traditional eradication measures yield limited results, it is necessary to develop more sustainable management approaches. Complete eradication of the blackchin tilapia is virtually impossible due to the fish’s high adaptability and reproductive potential [4]. One approach that has garnered attention is to produce high-value products from invasive species, transforming ecological burdens into economic opportunities [5]. However, such valorization should be integrated with invasive-species control measures and should not create incentives for the propagation or intentional spread of blackchin tilapia. Fish by-products such as heads, viscera, bones, and skin are sources of numerous bioactive compounds, including collagen, biocalcium, and oils rich in omega-3 fatty acids [6,7]. Previous studies on blackchin tilapia have mainly focused on the processing and quality attributes of fish flesh and surimi products [8], whereas information on the lipid composition and nutritional quality indices of oils recovered from its processing by-products remains limited. Due to its rapid spread, ecological risks, and limited economic utilization, transforming this fish into a nutritious source of fat could yield academic, economic, and social benefits.

Fish oil is an important dietary source of long-chain polyunsaturated fatty acids (PUFAs), particularly eicosapentaenoic acid (EPA) and docosahexaenoic acid (DHA), which have reported cardiovascular, neural, and anti-inflammatory benefits [9,10]. Expanding fish oil production to include alternative and underutilized feedstocks would support more resilient and sustainable use of aquatic resources [11,12]. The distribution of fats and fatty acids in fish is highly uneven across different parts of the body. For example, Selmi et al. [13] reported that the liver and entrails of the little tunny (*Euthynnus alletteratus*) contain more fat than muscle and exhibit different omega-3 fatty acid profiles. Similarly, Pudtikajorn and Benjakul found that the oil from the eyeballs of skipjack tuna has exceptionally high levels of DHA and EPA [14]. Specifically for tilapia, He et al. [7] reported that the viscera and head have higher fat contents than muscle. Importantly, fatty acid levels expressed as percentages of total fatty acids do not reflect the absolute fatty acid content per unit of tissue. Makhutova and Stoyanov [15], for example, reported both fatty acid levels (% of total fatty acids) and fatty acid contents (mg/g wet weight) across different tissues of Baikal grayling (*Thymallus baicalensis*), indicating that differences in total lipid content among tissues can influence the absolute amounts of EPA and DHA. Thus, both lipid content and fatty acid composition should be considered when evaluating fish by-products as potential sources of long-chain omega-3 fatty acids.

In addition to its fatty acid composition, the sensory quality of fish oil also depends significantly on the volatile substances produced from the oxidation and enzymatic breakdown of fats. These volatile compounds, which mainly consist of aldehydes, ketones, alcohols, and hydrocarbons, play a significant role in the aroma, taste, and consumer acceptance of fish oil [16]. Similarly to fats, the distribution of volatile compounds varies significantly across tissues, with viscera and trimmings being particularly susceptible to oxidation and self-degradation. Due to the presence of oxidation-catalyzing enzymes (e.g., lipase and phospholipase), there are high levels of iron from hemoglobin and phospholipids, while muscle and roe have different structural susceptibility to oxidation [17]. Previous research has also shown that oils obtained from different parts of the fish body differ in their fatty acid composition as well as their stability against oxidation. There is also increasing evidence of differences in sensory characteristics [18,19]. Therefore, analysis of the volatile compound profile provides valuable insights into the quality, safety, and application potential of oils extracted from invasive or underutilized fish species.

This study characterized the fatty acid composition, nutritional quality indices, volatile composition, and lipid oxidation status of oils extracted from blackchin tilapia by-products, including the head, viscera, roe, and mixed parts. Under the sampling conditions used in this study, the findings provide insights into the tissue-specific characteristics of these oils and their potential as lipid feedstocks for subsequent food, feed, or other lipid-based applications, supporting the valorization of processing by-products from this invasive species.

## 2. Materials and Methods

### 2.1. Sample Preparation and Oil Extraction

Blackchin tilapia by-products were obtained from fish weighing approximately 5–6 individuals per kilogram during a single fishing event in June 2025 in Samut Sakhon Province, Thailand. Fish were selected based on size, without regard to sex, and were harvested by local communities for household consumption and local food production. During processing, roe was separated when present.

The by-products were collected in two groups. For the individual-fraction group, the head, viscera, and roe were separated and collected individually until sufficient quantities were obtained. For the mixed-part group, by-products from subsequent fish were collected without separating the head, viscera, and roe. Thus, the resulting mixed fraction retained its approximate proportions of 79%, 16%, and 5% (wet weight), respectively, as generated during processing. For each by-product fraction, the collected material was divided into three separate bags, with each bag representing one independent sample replicate (*n* = 3). The collected by-products were washed thoroughly with tap water to remove surface impurities, sealed in aluminum foil bags, and stored at −18 °C until oil extraction.

For oil extraction, fish by-products were ground into small pieces and dried at 60 °C for 48 h, or until the moisture content was reduced to less than 10% (wet basis). Each sample was dried and extracted separately. The dried samples were extracted using the Soxhlet method with n-hexane as the solvent, following the method of Pinela et al. [20] with minor modifications. In summary, approximately 10 g of dry sample was placed in a cellulose extraction sheath and extracted with 150 mL of n-hexane for 6 h, requiring approximately 10–12 extraction cycles. After extraction, the solvent was removed under reduced pressure using a vacuum rotary evaporator (Büchi Vacobox B–177, BÜCHI Labortechnik AG, Flawil, Switzerland) at 40 °C. The extracted oil was weighed to calculate the extraction yield and stabilized with 200 ppm of butylated hydroxytoluene (BHT). The oil samples were stored in amber glass bottles under a nitrogen atmosphere at −20 °C until further analysis.

### 2.2. Determination of the Oil Yield

The yield of crude fish oil from four types of blackchin tilapia by-products was calculated as a percentage of the amount of oil extracted from the by-products using the following equation [21]:

Oil yield (%) = Extracted fish oil (g)/Weight of dry fish by-product sample (g) × 100(1)

### 2.3. Determination of the Fatty Acid Composition by Gas Chromatography with a Flame Ionization Detector (GC-FID)

The fatty acid composition was analyzed by GC-FID after converting oil to fatty acid methyl esters (FAMEs). Approximately 25 mg of oil sample was weighed into a 10 mL screw-capped tube, and 1.5 mL of 0.5 M methanolic sodium hydroxide was added. The headspace was flushed with nitrogen for 30 s, sealed, and heated at 80–100 °C for 2 min with intermittent shaking. After allowing it to cool to room temperature, methyl heptadecanoate (C17:0; 1 mL) was added as an internal standard, followed by 2 mL of boron trifluoride in methanol. The tube was again flushed with nitrogen, sealed, and heated at 80–100 °C for 30 min, then cooled. Iso-octane (1 mL) was added and mixed, followed by saturated sodium chloride solution (5 mL) to promote phase separation. The upper organic layer was collected, and a second extraction was performed with 2 mL of iso-octane. The combined extracts were evaporated under nitrogen, reconstituted in 1 mL of iso-octane, and transferred to GC vials.

FAME analysis was performed using a GC-FID instrument (Agilent 8890, Agilent Technologies, Santa Clara, CA, USA) equipped with a CP-Sil 88 column (100 m × 0.25 mm internal diameter, 0.20 μm). The injector and detector temperatures were set at 250 and 240 °C, respectively. The split ratio was 50:1. Helium was used as the carrier gas at a flow rate of 0.8 mL min^−1^. The oven temperature was programmed from 70 to 160 °C at 5 °C min^−1^ (18 min hold), then to 240 °C at 3 °C min^−1^ (20 min hold). Fatty acids were identified by comparison with a Supelco 37 Component FAME Mix (Supelco, Sigma-Aldrich, St. Louis, MO, USA) and quantified relative to the internal standard.

### 2.4. Determination of the Nutritional Quality Indices (NQI)

The NQI of blackchin tilapia oils from different by-products was calculated as follows [22]. UFA and FA denote unsaturated fatty acids and total fatty acids, respectively.

Index of atherogenicity (IA) = [C12:0 + (4 × C14:0) + C16:0)]/ΣUFA(2)

Index of thrombogenicity (IT) = (C14:0 + C16:0 + C18:0)/[(0.5 × ΣMUFA) + (0.5 × Σn − 6 PUFA) + (3 × Σn − 3 PUFA) + (n − 3/n − 6)](3)

Hypocholesterolemic/hypercholesterolemic (HH) ratio = (cis − C18:1 + ΣPUFA)/(C12:0 + C14:0 + C16:0)(4)

Health-promoting index (HPI) = ΣUFA/[C12: 0 + (4 × C14:0) + C16:0](5)

Fish lipid quality (FLQ) = 100 × (C22:6n − 3 + C20:5n − 3)/ΣFA(6)

Polyunsaturated fatty acid/saturated fatty acid ratio (PUFA/SFA) = ΣPUFA/ΣSFA(7)

Linoleic acid/α–linolenic acid ratio (LA/ALA) = C18:2n-6/C18:3n-3(8)

### 2.5. Analysis of Lipid Oxidation Indices

The peroxide value (PV) and the p-anisidine value (p-AV) were determined according to AOCS Official Methods Cd 8b-90 and Cd 18-90, respectively [23]. To determine the PV, the oil samples were dissolved in a chloroform–acetic acid mixture and reacted with a saturated potassium iodide solution; the liberated iodine was titrated according to the AOCS procedure. Total oxidation (TOTOX) was calculated as follows [24].

Total oxidation (TOTOX) = (2 × PV) + p-AV(9)

### 2.6. Evaluation of the Volatile Compound and Odor Profiles Using a Gas Chromatograph–Electronic Nose (GC-E-Nose)

The volatile compounds and odor profile of the crude oils extracted from different blackchin tilapia by-products were analyzed using a Heracles II electronic nose (Alpha M.O.S., Toulouse, France) equipped with dual capillary columns (MXT-5 and MXT-1701; 10 m × 180 µm × 0.4 µm, non-polar and polar, respectively) coupled with flame ionization detectors (FIDs), following the method of Banpacha et al. [25] with slight modifications. Briefly, 2.00 g of crude oil was transferred into a 20 mL headspace vial and sealed with a Teflon-faced silicone septum. Samples were equilibrated at 60 °C for 20 min under continuous agitation (500 rpm). Subsequently, 5 mL of headspace gas was injected via an automated headspace sampler at a flow rate of 125 µL/s, with the injector maintained at 200 °C. The trap temperature was set at 20 °C, and the split flow rate was set at 10 mL/min. The oven temperature was programmed to increase from 50 °C to 80 °C at 1 °C/s, and then to 250 °C at 3 °C/s, with a final holding time of 21 s. The FID temperature was maintained at 260 °C. Retention times were converted to Kovats retention indices using a standard mixture of n-alkanes (n-butane to n-hexadecane). Volatile compounds were tentatively identified by comparing their Kovats retention indices with the AroChemBase database integrated in AlphaSoft version 14.2 (Alpha M.O.S.). Only compounds with a library matching score greater than 80% were included in the subsequent analysis. Odor descriptors associated with the tentatively identified compounds were obtained from the AroChemBase database. Relative peak areas (%) obtained from the MXT-5 column chromatograms were used to compare the abundance of volatile compounds among the samples.

### 2.7. Statistical Analysis

Three independent sample replicates were analyzed for each by-product fraction (*n* = 3). The experimental data were evaluated by one-way analysis of variance (ANOVA), followed by Tukey’s honestly significant difference (HSD) test to compare the means. SPSS Statistics version 24 (SPSS Inc., Armonk, NY, USA) was used for all analyses, with *p* < 0.05 considered to indicate a statistically significant difference.

## 3. Results and Discussion

### 3.1. Oil Yield

The oil yield from various blackchin tilapia by-products is summarized in Table 1. The head had the highest oil yield (17.46%), followed by mixed parts (12.94%) and viscera (12.43%), which did not differ significantly from each other, whereas roe had the lowest oil yield (8.21%). The relatively high oil yield from the head may reflect the presence of lipid-containing tissues associated with the cranial region. Similar trends have been reported in other fish species, where the head provides a relatively high amount of recoverable oil compared with filleted flesh [26,27]. This finding highlights the potential of the head by-product as a raw material for oil recovery.

Although the viscera yielded less oil than the head, it still provided a relatively high amount of extractable oil, which is consistent with previous reports indicating that tilapia viscera typically contain around 13–14 g of fat per 100 g of sample [7]. For the mixed parts, the moderate oil yield found may reflect a combination of fatty components (heads and viscera) along with roe, resulting in an overall intermediate yield. Conversely, the roe yielded the least oil (8.21%), which may be related to the reproductive stage of the fish studied. The amount of fat accumulated in the reproductive glands of freshwater and brackish water fish is often highly variable and generally lower than in marine fish, depending on the physiological conditions and the spawning cycle of the fish.

### 3.2. Fatty Acid Composition

The fatty acid composition of oils extracted from various anatomical parts of blackchin tilapia is shown in Table 2. Overall, saturated fatty acids (SFAs) were the dominant group, followed by monounsaturated fatty acids (MUFAs) and PUFAs. Viscera oil showed the highest SFA level (58.83%), whereas mixed-part oil showed an intermediate value (56.20%), which is largely due to palmitic acid (C16:0), the major structural fatty acid in many fish [27]. The head had a slightly lower SFA level (55.74%) than viscera, but SFA remained the predominant fatty acid group in both tissues. This pattern is consistent with reports in several teleost species, which indicate that the head and viscera serve as important storage and structural components of fat [28].

In contrast, roe showed significantly higher levels of unsaturation, with roe having the highest PUFA level (14.84%) and the highest PUFA/SFA ratio (0.288). The higher PUFA level observed in roe may be associated with the physiological demands of reproduction, as long-chain ω-3 PUFAs are important structural components during early development. Long-chain ω-3 PUFAs, especially EPA (C20:5 n-3) and DHA (C22:6 n-3), are important for fish development during early life stages [29] and are abundant in fish roe [30]. Similar high levels of ω-3 PUFAs have also been reported in the roe of *Silurus glanis* [31].

As shown in Table 2, EPA and DHA were detected in all tissue-derived oils, with DHA consistently more abundant than EPA. The relative DHA level was highest in roe oil (7.45%), followed by mixed-part oil (4.78%), while head and viscera oils showed lower and statistically similar levels (4.16% and 3.60%, respectively). In contrast, EPA level was significantly lower in viscera oil (2.25%) than in head (2.88%), roe (3.02%), and mixed-part oils (3.01%), which did not differ significantly. The predominance of DHA over EPA is consistent with previous reports for fish roe and head oils [26,30]. The relatively high DHA and PUFA levels, together with the higher PUFA/SFA ratio, contributed to the relatively more favorable fatty acid profile of roe oil among the tissue-derived oils examined.

These differences in fatty acid composition may reflect the specific metabolic functions of each tissue, with the head and viscera serving primarily as storage and structural components of fat. By contrast, the roe is a highly metabolically active tissue rich in PUFAs that are important for reproductive processes. The results indicate that while the head and viscera of blackchin tilapia are useful for obtaining practical oil yields, the roe oil offers relatively more favorable nutritional characteristics, indicating that roe oil may warrant further investigation as a lipid resource for food and other value-added applications.

### 3.3. NQI of the Extracted Oils

Table 3 summarizes the nutritional quality indices (NQIs) of oils extracted from the head, viscera, roe, and mixed parts of blackchin tilapia. The PUFA/SFA ratio reflects the balance between fatty acids generally considered beneficial for cardiovascular health and those less desirable when consumed in excess [32]. In the present study, the PUFA/SFA ratios ranged from 0.146 in the viscera oil to 0.288 in the roe oil. Roe oil exhibited a significantly higher PUFA/SFA ratio than the other tissues (*p* < 0.05), consistent with its higher PUFA content and lower SFA proportion (Table 2). The relatively low PUFA/SFA ratios mainly reflect the predominance of palmitic acid (approximately 31–33% of total fatty acids), which accounted for the largest proportion of the SFA fraction in all tissues. Freshwater fish often contain lower proportions of long-chain PUFAs than many marine species, although variation in fatty acid composition can occur within and among fish species due to differences in diet, environmental conditions, and physiological status. Consequently, the PUFA/SFA ratios obtained in this study were lower than those commonly reported for marine fish oils. Chen and Liu [22] reported PUFA/SFA ratios of 0.42–2.12 for seaweed, 0.50–1.62 for fish, and 0.20–2.10 for shellfish, whereas Rahman et al. [33] reported values of 0.498–2.25 for Pangus fish oil, depending on the extraction method.

The n-3/n-6 ratio is an indicator reflecting the balance between omega-3 and omega-6 fatty acids. The n-3/n-6 ratios ranged from 2.917 to 3.401, with no significant differences among tissues (*p* > 0.05). The relatively high n-3/n-6 ratios were associated with the presence of EPA and DHA, together with the low linoleic acid levels observed across all tissues. The ratios obtained in this study were higher than those reported for tilapia (1.21–1.61), Pangus fish (0.010–0.398), and iridescent shark (0.24), and even exceeded those reported for rainbow trout (2.06) [7,33,34]. However, they remained lower than those of several marine fish species, which typically contain considerably greater amounts of long-chain omega-3 fatty acids, with reported n-3/n-6 ratios of 6.42 in herring, 4.97 in tuna, 12.6 in pollock, and 8.31 in Alaska pollock [34]. The linoleic acid/α-linolenic acid (LA/ALA) ratio ranged from 2.744 in the roe oil to 3.698 in the head oil, with no significant differences among tissues (*p* > 0.05). The relatively lower LA/ALA ratio of roe oil indicates a comparatively greater contribution of ALA relative to LA, although the differences among tissues were not statistically significant.

The index of atherogenicity (IA) and index of thrombogenicity (IT) are commonly used as indicators to characterize the relative contribution of fatty acids associated with atherogenic and thrombogenic potential [35]. In the present study, IA and IT ranged from 1.362 to 2.034 and 0.821 to 1.323, respectively. Roe oil exhibited lower IA and IT values than the other tissues. Lower IA and IT values reflect a lower relative contribution of the fatty acids included in the respective index calculations and are generally interpreted as indicating a more favorable fatty acid profile [36]. The relatively high IA values are attributable to the predominance of saturated fatty acids, particularly palmitic acid, in all oils. Compared with previous reports, the IA values were higher than those reported for Pangus fish oil (0.807) and common vegetable oils such as sunflower oil (0.09–0.11), but considerably lower than those of coconut oil (13.63) [22,33,37]. Likewise, the IT value of roe oil was within the upper range reported for fish (0.14–0.87) and comparable to those observed in several meat products [22]. These findings suggest that roe oil had the lowest values among the tissue-derived oils examined in this study, consistent with its higher proportion of PUFA and lower proportion of SFA.

The hypocholesterolemic/hypercholesterolemic ratio (HH) and health-promoting index (HPI) were also used to evaluate the nutritional quality of the extracted oils. HH and HPI values ranged from 0.485 to 0.671 and 0.492 to 0.734, respectively. The roe oil exhibited the highest HH and HPI values, with significantly higher HH than viscera oil and significantly higher HPI than all other oils. Higher HH values indicate a greater proportion of fatty acids associated with a more favorable cholesterol-related fatty acid profile, whereas higher HPI values reflect a more desirable fatty acid composition with respect to cardiovascular health [37]. However, HH is a compositional index derived from fatty acid profiles and does not directly demonstrate a cholesterol-lowering effect. The HH values obtained in this study (0.485–0.671) were below the range reported in previous fish studies (0.87–4.83) [22]. The HH and HPI values were lower than those reported for Pangus fish oil (HH = 3.703; HPI = 1.252) [33]. These results are consistent with the relatively low PUFA/SFA ratios observed in the present study and reflect the relatively high contribution of SFA to the overall fatty acid composition.

The fish lipid quality (FLQ) index ranged from 5.854 to 10.472, with roe oil showing a significantly higher value than the other tissues (*p* < 0.05). Because FLQ is calculated from the combined relative proportions of EPA and DHA, the higher FLQ value of roe oil reflects its greater relative contribution of these long-chain omega-3 fatty acids. The FLQ values obtained in this study were higher than those reported for Pangus fish oil (0.329–1.686) [33], but lower than the range reported for fish (13.01–36.37) [22]. These differences should be interpreted cautiously because FLQ is based on fatty acid composition and can vary substantially among species and tissues.

Overall, these nutritional quality indices indicate that roe oil had relatively more favorable lipid characteristics among the evaluated blackchin tilapia by-products, as evidenced by its higher PUFA/SFA, HH, HPI, and FLQ values, together with its lowest IA and IT values. Although the nutritional quality of these oils may be lower than that of marine fish oils, blackchin tilapia by-products could be further investigated as potential lipid feedstocks for food, feed, or other lipid-based applications, subject to appropriate refining, stabilization, safety, and contaminant assessment.

### 3.4. Lipid Oxidation Indices of the Extracted Oils

The oxidation indices (Table 4) revealed marked differences among tissues. Head oil showed the highest PV, p-AV, and TOTOX values (58.16 ± 5.74 meq O_2_/kg, 33.58 ± 2.17, and 149.90 ± 13.52, respectively), whereas roe oil showed the lowest values (5.28 ± 0.10 meq O_2_/kg, 5.02 ± 0.30, and 15.59 ± 0.49, respectively). The higher PV in head oil indicates greater accumulation of primary lipid oxidation products, while the higher p-AV reflects greater accumulation of secondary oxidation products, particularly aldehydic compounds. Accordingly, the TOTOX value, which combines primary and secondary oxidation indices, was substantially higher in head oil than in the other tissue-derived oils. In contrast, roe oil exhibited the lowest values for all three indices, indicating a comparatively lower degree of lipid oxidation under the conditions examined.

Although roe oil contained the highest proportion of PUFA, including DHA, it showed the lowest oxidation indices, whereas head oil showed substantially higher values despite its lower PUFA proportion. This suggests that oxidation was not determined solely by PUFA content. Previous research on n-3 PUFA supplements has similarly shown that oxidation markers did not differ significantly among products with different EPA+DHA concentrations, indicating that factors beyond the amount of n-3 PUFA may contribute to the observed oxidation status [38]. Jairoun et al. [24] reported mean PV, p-AV, and TOTOX values of 6.4, 11.0, and 23.8, respectively, in 44 commercial fish-oil supplements. Although these values provide a useful reference, direct comparison with the present crude oils should be made cautiously because of differences in sample type and processing history.

The oxidation indices were broadly consistent with the volatile profiles, as head oil showed relatively high levels of 2-hexanol and propanal, whereas roe oil showed lower oxidation indices and a distinct volatile profile. However, individual volatile compounds may originate from multiple biochemical and processing pathways. In addition, the drying treatment at 60 °C for 48 h may have influenced the oxidation status of the recovered oils. Therefore, the results represent the oxidation status of the n-hexane-extractable crude oils after the applied pretreatment rather than the intrinsic oxidation state of the fresh tissues.

### 3.5. Volatile Compound Profiles and Odor Characteristics of the Extracted Oils

The volatile compound profiles of the crude oils extracted from the head, viscera, roe, and mixed by-products of blackchin tilapia are summarized in Table 5. Twenty-two major volatile compounds were tentatively identified and classified into aldehydes, ketones, alcohols, sulfur compounds, hydrocarbons, and other compounds. There were marked differences in the volatile compound composition among the four crude oils, suggesting that tissue origin may contribute to differences in their potential odor characteristics. Sulfur compounds and alcohols represented the predominant volatile compound groups, whereas hydrocarbons and other minor compounds had comparatively lower abundances (Table 5).

Among the identified compounds, 1-propanethiol was the predominant tentatively identified volatile compound in all crude oils, accounting for 30.88%, 37.26%, 84.22%, and 68.69% of the relative peak area in the head, viscera, roe, and mixed-part oils, respectively. Other sulfur-containing compounds, including dimethyl disulfide, 2-methylthiophene, and 3-methyl-3-sulfanylbutan-1-ol, were detected at considerably lower abundances. Sulfur-containing volatile compounds in seafood can arise from the degradation of sulfur-containing amino acids and other sulfur-containing precursors through postmortem biochemical reactions and thermal processing [39]. Although the exact pathway responsible for the formation of 1-propanethiol in the present study remains unclear, the high abundance of 1-propanethiol in the roe oil may be associated with the availability of sulfur-containing precursors in roe. Fish roe is rich in proteins and free amino acids, including methionine [40,41], and methionine can contribute to the formation of sulfur-containing volatile compounds through amino acid degradation pathways [39]. Importantly, the by-products were dried at 60 °C for 48 h before oil extraction. This pretreatment may have altered the volatile profile by promoting the formation, transformation, or release of volatile sulfur compounds. However, the present study did not measure sulfur-containing amino acids before and after drying. Therefore, it cannot confirm whether these precursors were degraded during drying or how much drying contributed to 1-propanethiol formation. The high relative abundance of 1-propanethiol detected in roe oil may reflect both tissue-derived precursors and changes associated with sample pretreatment.

From a sensory perspective, sulfur-containing compounds can have extremely low odor thresholds and may contribute substantially to aroma perception even when present at relatively low concentrations [42]. The predominance of 1-propanethiol, together with the presence of dimethyl disulfide and 2-methylthiophene, suggests that these sulfur-containing volatile compounds may contribute to the sulfurous odor characteristics of the crude roe oil. Given that the oils obtained in this study were analyzed in their crude state without refining or deodorization, these endogenous sulfur-containing volatile compounds may have been retained and may contribute to the overall volatile compound profile.

The head oil exhibited the highest abundance of alcohols, particularly 2-hexanol (23.64%), together with relatively high levels of 2,3-butanediol and heptan-2-ol. In addition, propanal was detected exclusively in the head oil (13.95%), whereas acetaldehyde and 2-methylbutanal were present at lower levels. Alcohols can be formed from lipid-derived hydroperoxides and carbonyl compounds during lipid oxidation, whereas propanal is considered a secondary oxidation marker associated with n-3 PUFAs [16,43]. In contrast, typical oxidation-derived aldehydes such as hexanal were not detected. The absence of hexanal may indicate limited formation or accumulation of this oxidation product under the extraction and storage conditions used in the present study. The addition of BHT after extraction may have further limited oxidative changes during sample handling and storage. This interpretation should be considered together with the oxidation indices, which provide additional evidence regarding the oxidative status of the extracted oils. Therefore, the volatile compounds detected in the present study cannot be assumed to originate exclusively from the original tissues, because the drying and Soxhlet extraction conditions may have contributed to lipid oxidation and thermal formation of some volatile compounds.

The volatile compound composition of the head oil also suggests a different odor profile from that of the roe oil. The relatively high abundance of 2-hexanol, described as fatty, fruity, and green, together with 2,3-pentanedione, described as buttery, creamy, and caramel-like, may contribute to the corresponding odor characteristics of the head oil. In contrast, the lower abundance of sulfur-containing compounds in the head oil may reduce the intensity of sulfurous notes compared with the roe oil. However, the sensory contribution of individual volatile compounds depends not only on their abundance but also on their odor thresholds and interactions among volatile constituents [42]. The viscera oil exhibited comparatively higher abundances of 2-methylbutanal, cyclohexanone, (S)-2-methyl-1-butanol, 3-methyl-3-sulfanylbutan-1-ol, and several hydrocarbons, suggesting a more diverse volatile compound profile. The mixed-part oil displayed an intermediate volatile compound profile, reflecting the combined contribution of multiple tissue types rather than the predominance of a single group of volatile compounds.

To facilitate comparison of the overall volatile compound patterns, the relative peak areas were standardized by row-wise Z-score transformation and visualized as a heatmap (Figure 1). Unlike Table 5, which presents quantitative differences in individual volatile compounds, the heatmap emphasizes similarities and differences in volatile fingerprints among the tissue-derived oils after normalization of each compound.

The relatively similar patterns among replicates within each tissue suggest reproducible tissue-associated volatile fingerprints under the conditions examined. More importantly, there were clear tissue-dependent patterns. The head oil was characterized by enrichment of aldehydes, alcohols, and 2,3-pentanedione, whereas the roe oil exhibited distinct enrichment of sulfur-containing compounds, particularly 1-propanethiol. The viscera oil showed relatively higher abundances of several minor ketones, alcohols, hydrocarbons, and miscellaneous compounds, while the mixed-part oil displayed an intermediate volatile fingerprint consistent with its origin from multiple tissues. These observations are in good agreement with the quantitative results presented in Table 5 and support the presence of tissue-dependent volatile fingerprints.

### 3.6. Limitations and Future Perspectives

Several limitations should be considered when interpreting the present findings. First, the samples were collected during a single fishing event in June 2025, and the sex and reproductive maturity of the fish were not recorded. Therefore, seasonal and physiological variations, particularly those associated with the reproductive cycle, could not be assessed. Consequently, whether the oil yield and fatty acid composition of roe oil would remain consistent across different sampling periods cannot be determined from the present study, and the nutritional characteristics reported here should be interpreted as representative of the sampled period rather than generalized across seasons or reproductive stages. Second, the oils were recovered using n-hexane Soxhlet extraction after drying at 60 °C for 48 h. Compared with comprehensive lipid extraction methods such as the Folch method, n-hexane may recover non-polar lipids and may not quantitatively extract polar lipid fractions. Therefore, the reported yields represent n-hexane-extractable crude oils, and differences in the relative recovery of lipid classes among tissues may have influenced the observed fatty acid profiles. In addition, the drying pretreatment may have influenced lipid oxidation and volatile characteristics, including the formation, transformation, or release of volatile compounds. Because sulfur-containing precursors were not quantified before and after drying, the specific contribution of the pretreatment to the formation of 1-propanethiol could not be determined. Contaminant levels, storage stability, refining requirements, and application-specific safety were not evaluated. Future studies should therefore include broader sampling across seasons and reproductive stages, comparison of extraction and pretreatment approaches, and evaluation of safety, storage stability, and downstream processing requirements before practical application of these oils.

## 4. Conclusions

Oil yield and lipid characteristics varied among the different by-product fractions examined. The head provided the highest oil yield, whereas roe oil showed a relatively more favorable fatty acid profile among the tissue-derived oils examined, characterized by higher levels of PUFA and long-chain omega-3 fatty acids and more favorable nutritional quality indices. However, its nutritional characteristics were still less favorable than those reported for several marine fish oils, as indicated by its relatively low PUFA/SFA and HH values and lower relative abundance of long-chain omega-3 fatty acids. The crude oils also exhibited distinct lipid oxidation and volatile profiles among tissues. Head oil showed the highest levels of primary and secondary oxidation products, whereas roe oil showed the lowest oxidation indices. The volatile profiles were tissue-dependent, with 1-propanethiol predominating in roe oil and 2-hexanol being relatively abundant in head oil. In conclusion, these findings highlight the importance of tissue-specific selection when recovering oils from blackchin tilapia processing by-products and support their potential valorization as sources of oils with distinct lipid and volatile characteristics.

## Figures and Tables

**Figure 1:**
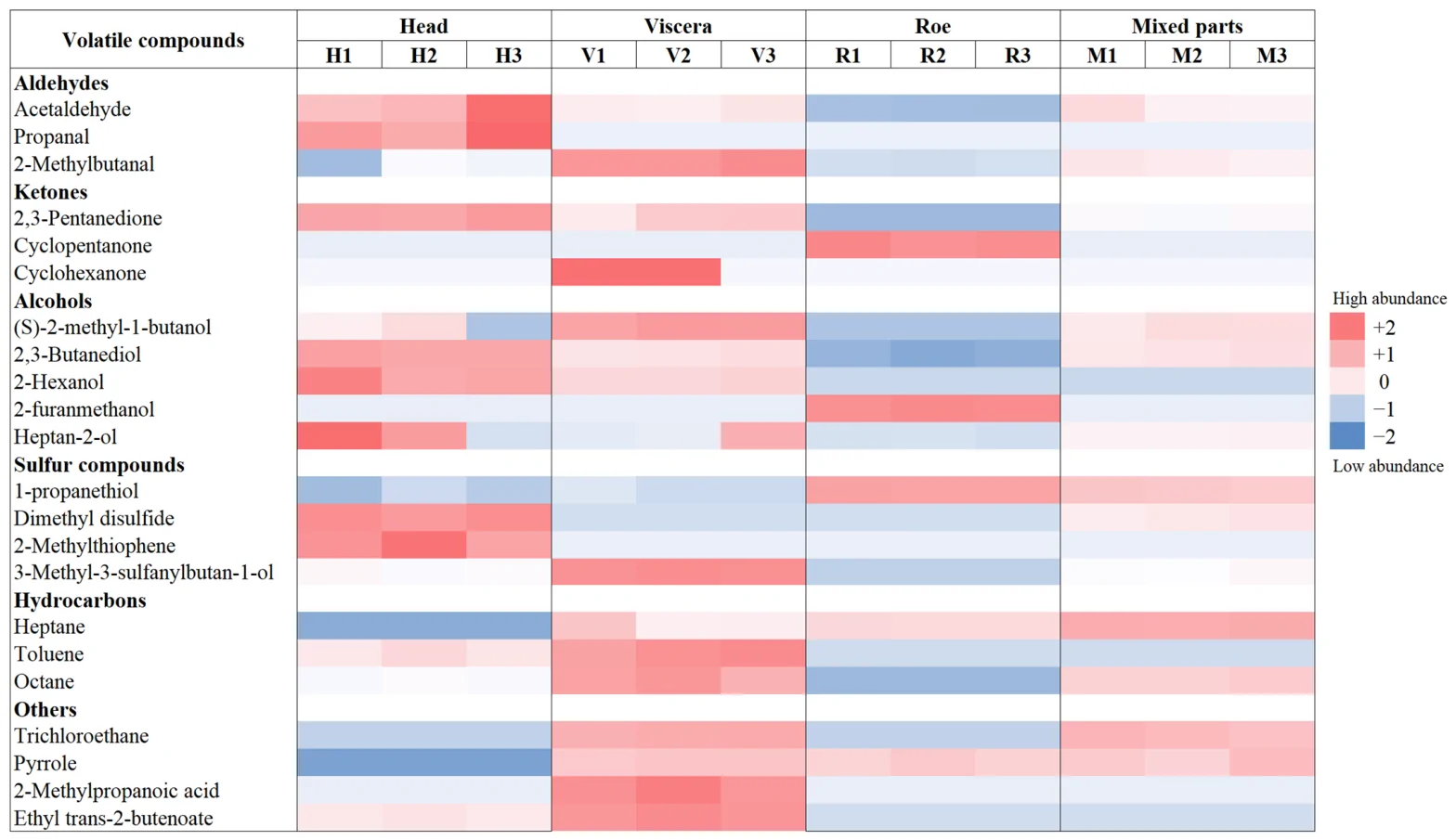
Heatmap of row-wise Z-score-transformed relative peak areas of volatile compounds in oils extracted from blackchin tilapia head, viscera, roe, and mixed by-products. Rows represent volatile compounds, and columns represent independent sample replicates (*n* = 3).

**Table 1:** Extraction yield of oils extracted from different parts of blackchin tilapia by-products.

Parts	Oil Yield (% Dry-Weight Basis)
Head	17.46 ± 1.44 ^a^
Viscera	12.43 ± 1.06 ^b^
Roe	8.21 ± 0.79 ^c^
Mixed parts	12.94 ± 0.21 ^b^

Note: Data are presented as mean ± SD. Different superscript letters in the same column indicate significant differences (*p* < 0.05).

**Table 2:** Fatty acid composition (% of total fatty acids) of oils extracted from different parts of blackchin tilapia by-products.

Fatty Acid (% of Total Fatty Acids)	Head	Viscera	Roe	Mixed Parts
Lauric acid (C12:0)	0.12 ± 0.04 ^a^	0.10 ± 0.01 ^a^	0.07 ± 0.04 ^a^	0.11 ± 0.00 ^a^
Tridecanoic acid (C13:0)	0.10 ± 0.02 ^a^	0.10 ± 0.03 ^a^	0.05 ± 0.01 ^a^	0.09 ± 0.01 ^a^
Myristic acid (C14:0)	12.88 ± 0.06 ^a^	12.70 ± 0.07 ^a^	8.66 ± 0.41 ^b^	11.95 ± 0.82 ^a^
Pentadecanoic acid (C15:0)	3.58 ± 0.02 ^a^	3.68 ± 0.11 ^a^	1.98 ± 0.22 ^b^	3.17 ± 0.10 ^a^
Palmitic acid (C16:0)	31.51 ± 1.08 ^a^	32.79 ± 1.20 ^a^	31.15 ± 1.27 ^a^	32.22 ± 0.96 ^a^
Stearic acid (C18:0)	5.51 ± 0.11 ^c^	7.28 ± 0.26 ^a^	6.35 ± 0.17 ^b^	6.30 ± 0.14 ^b^
Arachidic acid (C20:0)	0.20 ± 0.02 ^a^	0.21 ± 0.07 ^a^	0.23 ± 0.02 ^a^	0.23 ± 0.00 ^a^
Behenic acid (C22:0)	0.09 ± 0.04 ^a^	0.11 ± 0.04 ^a^	0.12 ± 0.15 ^a^	0.12 ± 0.05 ^a^
Tricosanoic acid (C23:0)	1.67 ± 0.13 ^b^	1.77 ± 0.02 ^b^	2.95 ± 0.07 ^a^	1.93 ± 0.10 ^b^
Lignoceric acid (C24:0)	0.08 ± 0.01 ^a^	0.10 ± 0.00 ^a^	0.08 ± 0.04 ^a^	0.08 ± 0.03 ^a^
Saturated fatty acids (SFAs)	55.74 ± 0.77 ^b^	58.83 ± 1.09 ^a^	51.64 ± 0.49 ^c^	56.20 ± 0.10 ^ab^
Myristoleic acid (C14:1)	0.20 ± 0.01 ^a^	0.19 ± 0.02 ^ab^	0.15 ± 0.01 ^b^	0.18 ± 0.00 ^ab^
Palmitoleic acid (C16:1)	19.17 ± 0.54 ^ab^	18.16 ± 0.61 ^b^	20.99 ± 0.17 ^a^	19.32 ± 0.99 ^ab^
Elaidic acid (C18:1n9t)	0.23 ± 0.01 ^a^	0.25 ± 0.00 ^a^	0.23 ± 0.02 ^a^	0.25 ± 0.01 ^a^
Oleic acid (C18:1n9c)	13.31 ± 0.76 ^a^	13.37 ± 0.96 ^a^	11.90 ± 0.57 ^a^	12.82 ± 0.35 ^a^
Heneicosanoic acid (C20:1)	0.27 ± 0.01 ^ab^	0.32 ± 0.02 ^a^	0.19 ± 0.01 ^b^	0.22 ± 0.03 ^b^
Erucic acid (C22:1n9)	0.09 ± 0.03 ^a^	0.10 ± 0.02 ^a^	0.04 ± 0.01 ^a^	0.08 ± 0.01 ^a^
Nervonic acid (C24:1)	0.04 ± 0.01 ^a^	0.04 ± 0.01 ^a^	0.02 ± 0.01 ^a^	0.03 ± 0.02 ^a^
Monounsaturated fatty acids (MUFAs)	33.30 ± 0.27 ^a^	32.44 ± 0.33 ^a^	33.52 ± 0.43 ^a^	32.90 ± 0.57 ^a^
Linoleic acid (C18:2n6c)	1.85 ± 0.41 ^a^	1.40 ± 0.77 ^a^	1.85 ± 0.14 ^a^	1.72 ± 0.13 ^a^
γ -Linolenic acid (C18:3n6)	0.50 ± 0.03 ^b^	0.39 ± 0.03 ^c^	0.68 ± 0.01 ^a^	0.51 ± 0.02 ^b^
Linolenic acid (C18:3n3)	0.95 ± 0.10 ^a^	0.52 ± 0.09 ^c^	0.84 ± 0.02 ^ab^	0.60 ± 0.06 ^bc^
cis-8,11,14-Eicosatrienoic acid (C20:3n6)	0.45 ± 0.0 ^b^	0.43 ± 0.04 ^b^	0.83 ± 0.03 ^a^	0.54 ± 0.05 ^b^
cis-11,14,17-Eicosatrienoic acid (C20:3n3)	0.15 ± 0.00 ^a^	0.12 ± 0.00 ^a^	0.15 ± 0.00 ^a^	0.16 ± 0.09 ^a^
Arachidonic acid (C20:4n6)	0.02 ± 0.01 ^a^	0.03 ± 0.01 ^a^	0.01 ± 0.00 ^a^	0.03 ± 0.01 ^a^
cis-5,8,11,14,17-Eicosapentaenoic acid (C20:5n3)	2.88 ± 0.03 ^a^	2.25 ± 0.05 ^b^	3.02 ± 0.05 ^a^	3.01 ± 0.06 ^a^
cis-4,7,10,13,16,19-Docosahexaenoic acid (C22:6n3)	4.16 ± 0.02 ^c^	3.60 ± 0.06 ^c^	7.45 ± 0.02 ^a^	4.78 ± 0.28 ^b^
Polyunsaturated fatty acids (PUFAs)	10.96 ± 0.50 ^b^	8.73 ± 0.76 ^c^	14.84 ± 0.06 ^a^	10.91 ± 0.47 ^b^

Note: Data are presented as mean ± SD. Different superscript letters in the same row indicate significant differences (*p* < 0.05).

**Table 3:** Nutritional Quality Indices (NQI) of oils extracted from different parts of blackchin tilapia by-products.

NQI	Head	Viscera	Roe	Mixed Parts
PUFA/SFA	0.197 ± 0.012 ^b^	0.146 ± 0.016 ^c^	0.288 ± 0.004 ^a^	0.194 ± 0.008 ^b^
n-3/n-6	2.917 ± 0.402 ^a^	3.076 ± 1.065 ^a^	3.401 ± 0.132 ^a^	3.062 ± 0.035 ^a^
IA	1.879 ± 0.062 ^a^	2.034 ± 0.076 ^a^	1.362 ± 0.005 ^b^	1.830 ± 0.057 ^a^
IT	1.099 ± 0.027 ^b^	1.323 ± 0.016 ^a^	0.821 ± 0.018 ^c^	1.084 ± 0.009 ^b^
HH	0.546 ± 0.042 ^ab^	0.485 ± 0.049 ^b^	0.671 ± 0.030 ^a^	0.536 ± 0.020 ^ab^
HPI	0.533 ± 0.018 ^b^	0.492 ± 0.018 ^c^	0.734 ± 0.003 ^a^	0.547 ± 0.017 ^b^
LA/ALA	3.698 ± 0.596 ^a^	3.551 ± 1.690 ^a^	2.744 ± 0.171 ^a^	3.368 ± 0.136 ^a^
FLQ	7.042 ± 0.010 ^bc^	5.854 ± 0.106 ^d^	10.472 ± 0.070 ^a^	7.789 ± 0.221 ^b^

Note: Data are presented as mean ± SD. Abbreviations: IA: Index of atherogenicity, IT: Index of thrombogenicity, HH: Hypo and Hypercholesterolemia ratio, HPI: Health promoting index, FLQ: Fish lipid quality. Different lowercase letters within the same row indicate significant differences (*p* < 0.05).

**Table 4:** Lipid oxidation indices of crude oil from blackchin tilapia by-products.

Parts	PV (meq O_2_/kg)	p-AV	TOTOX
Head	58.16 ± 5.74 ^a^	33.58 ± 2.17 ^a^	149.90 ± 13.52 ^a^
Viscera	13.67 ± 0.14 ^c^	17.53 ± 1.19 ^c^	44.86 ± 1.44 ^c^
Roe	5.28 ± 0.10 ^d^	5.02 ± 0.30 ^d^	15.59 ± 0.49 ^d^
Mixed parts	47.65 ± 2.13 ^b^	22.43 ± 0.98 ^b^	117.72 ± 4.31 ^b^

Different lowercase letters within the same column indicate significant differences (*p* < 0.05).

**Table 5:** Major volatile compounds, relative peak areas (%), and odor characteristics of oils extracted from blackchin tilapia by-products.

Retention Time (min)	Volatile Compound	Odor Description	Relative Peak Area (%)			
			Head	Viscera	Roe	Mixed Parts
Aldehydes						
16.1	Acetaldehyde	Fruity, green, pungent	1.34 ± 0.40	Trace (<1%)	Trace (<1%)	Trace (<1%)
18.6	Propanal	Pungent, earthy, nutty	13.95 ± 3.97	ND	ND	ND
29.7	2-Methylbutanal	Malty, cocoa, nutty	3.12 ± 0.52	5.46 ± 0.12	3.03 ± 0.04	3.82 ± 0.11
Ketones						
33.0	2,3-Pentanedione	Buttery, creamy, caramel-like	8.64 ± 0.40	5.63 ± 1.14	ND	3.29 ± 0.20
43.0	Cyclopentanone	Minty	ND	ND	1.54 ± 0.07	ND
54.5	Cyclohexanone	Minty, acetone-like	ND	1.89 ± 1.63	ND	ND
Alcohols						
36.6	(S)-2-methyl-1-butanol	Fruity, Malty	Trace (<1%)	1.05 ± 0.04	ND	Trace (<1%)
42.1	2,3-Butanediol	Creamy, Fruity,	1.88 ± 0.04	1.22 ± 0.04	Trace (<1%)	1.19 ± 0.05
44.7	2-Hexanol	Fatty, Fruity, Green	23.64 ± 4.32	12.64 ± 0.52	ND	ND
50.5	2-furanmethanol	Caramel-like, coffee, sweet	ND	ND	1.59 ± 0.05	ND
54.4	Heptan-2-ol	Fatty, floral, fruity	2.79 ± 2.37	1.26 ± 1.46	Trace (<1%)	1.07 ± 0.04
Sulfur compounds						
27.0	1-propanethiol	Sulfurous, onion-like, cabbage-like	30.88 ± 4.99	37.26 ± 2.47	84.22 ± 0.07	68.69 ± 1.47
39.7	Dimethyl disulfide	Sulfurous, garlic, cabbage-like	1.15 ± 0.06	ND	ND	Trace (<1%)
43.4	2-Methylthiophene	Sulfurous, onion-like	1.04 ± 0.22	ND	ND	ND
60.2	3-Methyl-3-sulfanylbutanol-1-ol	Meaty, onion-like	Trace (<1%)	1.44 ± 0.02	ND	Trace (<1%)
Hydrocarbons						
34.4	Heptane	Gasoline-like	ND	3.10 ± 0.80	3.37 ± 0.05	4.88 ± 0.03
40.9	Toluene	Solvent-like	Trace (<1%)	2.27 ± 0.21	ND	ND
43.2	Octane	Gasoline-like	Trace (<1%)	2.38 ± 0.22	ND	1.64 ± 0.06
Others						
28.7	Trichloroethane	Ether-like	ND	4.79 ± 0.13	ND	4.20 ± 0.27
38.4	Pyrrole	Nutty, coffee-like	ND	1.35 ± 0.03	1.25 ± 0.06	1.32 ± 0.13
42.8	2-Methylpropanoic acid	Rancid, cheesy	ND	1.74 ± 0.19	ND	ND
48.1	Ethyl trans-2-butenoate	Sweet, fruity	Trace (<1%)	1.89 ± 0.09	ND	ND

Note: Values are presented as mean ± SD. ND: Not detected. Retention time based on MXT5 column. Odor description was adapted from the instrument library.

## Data Availability

The original contributions presented in this study are included in the article. Further inquiries can be directed to the corresponding author.

## References

[1] Gozlan R.E., Bommarito C., Caballero-Huertas M., Givens J., Mortillaro J.-M., Pepey E., Ralaiarison R.P., Senff P., Combe M. (2024). A One-Health approach to non-native species, aquaculture, and food security. Water Biol. Secur..

[2] Chaianunporn T., Panthum T., Singchat W., Chaianunporn K., Suksavate W., Chaiyes A., Muangmai N., Marod D., Duengkae P., Srikulnath K. (2024). Sustainable ecosystem management strategies for tackling the invasion of blackchin tilapia (*Sarotherodon melanotheron*) in Thailand: Guidelines and considerations. Animals.

[3] Panfili J., Mbow A., Durand J.-D., Diop K., Diouf K., Thior D., Ndiaye P., Laë R. (2004). Influence of salinity on the life-history traits of the West African black-chinned tilapia (*Sarotherodon melanotheron*): Comparison between the Gambia and Saloum estuaries. Aquat. Living Resour..

[4] Suwannarat N., Ribas A., Miquel J., Poonlaphdecha S. (2025). Helminth absence and invasion success of blackchin tilapia (*Sarotherodon melanotheron*) in Thailand. Front. Vet. Sci..

[5] Roy V.C., Islam M.R., Sadia S., Yeasmin M., Park J.-S., Lee H.-J., Chun B.-S. (2023). Trash to treasure: An up-to-date understanding of the valorization of seafood by-products, targeting the major bioactive compounds. Mar. Drugs.

[6] Waqar M., Sajjad N., Ullah Q., Vasanthkumar S.S., Ahmed F., Panpipat W., Aluko R.E., Kaur L., Chaijan M., Ageru T.A. (2025). Fish by-products utilization in food and health: Extraction technologies, bioactive, and sustainability challenges. Food Sci. Nutr..

[7] He C., Cao J., Bao Y., Sun Z., Liu Z., Li C. (2021). Characterization of lipid profiling in three parts (muscle, head and viscera) of tilapia (*Oreochromis niloticus*) using lipidomics with UPLC-ESI-Q-TOF-MS. Food Chem..

[8] Perdio A.C., Valerio R.B., Consigna M.J.S., Llave D.S., Gonzales A.J.V., Turtur M.L. (2022). Quality attributes of black-chin tilapia (*Sarotherodon melanotheron*) surimi treated with different cryoprotectants. Food Res..

[9] Manson J.E., Cook N.R., Lee I.-M., Christen W., Bassuk S.S., Mora S., Gibson H., Albert C.M., Gordon D., Copeland T. (2019). Marine n−3 fatty acids and prevention of cardiovascular disease and cancer. N. Engl. J. Med..

[10] Abdelhamid A.S., Brown T.J., Brainard J.S., Biswas P., Thorpe G.C., Moore H.J., Deane K.H., AlAbdulghafoor F.K., Summerbell C.D., Worthington H.V. (2018). Omega-3 fatty acids for the primary and secondary prevention of cardiovascular disease. Cochrane Database Syst. Rev..

[11] Tocher D., Betancor M., Sprague M., Olsen R., Napier J. (2019). Omega-3 long-chain polyunsaturated fatty acids, EPA and DHA: Bridging the gap between supply and demand. Nutrients.

[12] Rodrigues M., Rosa A., Almeida A., Martins R., Ribeiro T.Â., Pintado M., Gonçalves R.F.S., Pinheiro A.C., Fonseca A.J.M., Maia M.R.G. (2024). Omega-3 fatty acids from fish by-products: Innovative extraction and application in food and feed. Food Bioprod. Process..

[13] Selmi S., Mbarki R., Sadok S. (2008). Seasonal change of lipid and fatty acid composition of little tuna *Euthynnus alletteratus* by-products. Nutr. Health.

[14] Pudtikajorn K., Benjakul S. (2020). Simple wet rendering method for extraction of prime quality oil from skipjack tuna eyeballs. Eur. J. Lipid Sci. Technol..

[15] Makhutova O.N., Stoyanov K.N. (2021). Fatty acid content and composition in tissues of Baikal Grayling (*Thymallus baicalensis*), with a special focus on DHA synthesis. Aquac. Int..

[16] Shahidi F., Hossain A. (2022). Role of lipids in food flavor generation. Molecules.

[17] Nikoo M., Regenstein J.M., Vayghan A.H., Walayat N. (2023). Formation of oxidative compounds during enzymatic hydrolysis of byproducts of the seafood industry. Processes.

[18] Zhong Y., Madhujith T., Mahfouz N., Shahidi F. (2007). Compositional characteristics of muscle and visceral oil from steelhead trout and their oxidative stability. Food Chem..

[19] Monteiro J.P., Sousa T., Melo T., Pires C., Marques A., Nunes M.L., Calado R., Domingues M.R. (2024). Unveiling the lipid features and valorization potential of Atlantic salmon (*Salmo salar*) heads. Mar. Drugs.

[20] Pinela J., De La Fuente B., Rodrigues M., Pires T.C.S.P., Mandim F., Almeida A., Dias M.I., Caleja C., Barros L. (2023). Upcycling fish by-products into bioactive fish oil: The suitability of microwave-assisted extraction. Biomolecules.

[21] Nazir N., Diana A., Sayuti K. (2017). Physicochemical and fatty acid profile of fish oil from head of tuna (*Thunnus albacares*) extracted from various extraction method. Int. J. Adv. Sci. Eng. Inf. Technol..

[22] Chen J., Liu H. (2020). Nutritional indices for assessing fatty acids: A mini-review. Int. J. Mol. Sci..

[23] AOCS (1997). Official Methods and Recommended Practices of the American Oil Chemists’ Society.

[24] Jairoun A.A., Shahwan M., Zyoud S.H. (2020). Fish oil supplements, oxidative status, and compliance behaviour: Regulatory challenges and opportunities. PLoS ONE.

[25] Banpacha N., Janpeng H., Renaldi G., Samakradhamrongthai R.S. (2025). Influence of semi-fermentation and drying on the phytochemical properties, antioxidant activity, and volatile compounds of *Gymnema inodorum* (Lour.) Decne leaves. Appl. Food Res..

[26] Zhang J., Tao N., Zhao Y., Wang X., Wang M. (2019). Comparison of the fatty acid and triglyceride profiles of big eye tuna (*Thunnus obesus*), Atlantic salmon (*Salmo salar*) and bighead carp (*Aristichthys nobilis*) heads. Molecules.

[27] Kandyliari A., Mallouchos A., Papandroulakis N., Golla J.P., Lam T.T., Sakellari A., Karavoltsos S., Vasiliou V., Kapsokefalou M. (2020). Nutrient composition and fatty acid and protein profiles of selected fish by-products. Foods.

[28] Rohim A., Estiasih T., Susilo B., Nisa F.C. (2024). Extraction of healthy oils from fish viscera by conventional and advanced technologies. Grasas Aceites.

[29] Tocher D.R. (2015). Omega-3 long-chain polyunsaturated fatty acids and aquaculture in perspective. Aquaculture.

[30] Shirai N., Higuchi T., Suzuki H. (2006). Analysis of lipid classes and the fatty acid composition of the salted fish roe food products, ikura, tarako, tobiko and kazunoko. Food Chem..

[31] Saliu F., Leoni B., Della Pergola R. (2017). Lipid classes and fatty acids composition of the roe of wild *Silurus glanis* from subalpine freshwater. Food Chem..

[32] Hart T.L., Damani J.J., DiMattia Z.S., Tate K.E., Jafari F., Petersen K.S. (2025). Dietary Polyunsaturated to Saturated Fatty Acid Ratio as an Indicator for LDL Cholesterol Response: A Systematic Review and Meta-Analysis of Randomized Clinical Trials. Adv. Nutr..

[33] Rahman N., Hashem S., Akther S., Jothi J.S. (2023). Impact of various extraction methods on fatty acid profile, physicochemical properties, and nutritional quality index of Pangus fish oil. Food Sci. Nutr..

[34] Strobel C., Jahreis G., Kuhnt K. (2012). Survey of n-3 and n-6 polyunsaturated fatty acids in fish and fish products. Lipids Health Dis..

[35] Ulbricht T.L.V., Southgate D.A.T. (1991). Coronary heart disease: Seven dietary factors. Lancet.

[36] Karimian-Khosroshahi N., Hosseini H., Rezaei M., Khaksar R., Mahmoudzadeh M. (2015). Effect of different cooking methods on minerals, vitamins, and nutritional quality indices of Rainbow Trout (*Oncorhynchus mykiss*). Int. J. Food Prop..

[37] Chen S., Bobe G., Zimmerman S., Hammond E.G., Luhman C.M., Boylston T.D., Freeman A.E., Beitz D.C. (2004). Physical and sensory properties of dairy products from cows with various milk fatty acid compositions. J. Agric. Food Chem..

[38] Jackowski S.A., Alvi A.Z., Mirajkar A., Imani Z., Gamalevych Y., Shaikh N.A., Jackowski G. (2015). Oxidation levels of North American over-the-counter n-3 (omega-3) supplements and the influence of supplement formulation and delivery form on evaluating oxidative safety. J. Nutr. Sci..

[39] Varlet V., Fernandez X. (2010). Review. Sulfur-containing volatile compounds in seafood: Occurrence, odorant properties and mechanisms of formation. Food Sci. Technol. Int..

[40] Topuz O.K., Yatmaz H.A., Alp A.C., Kaya A., Yerlikaya P. (2021). Biogenic amine formation in fish roe in under the effect of drying methods and coating materials. J. Food Process. Preserv..

[41] Ren Z., Yang F., Yao S., Bi L., Jiang G., Huang J., Tang Y. (2022). Effects of low molecular weight peptides from monkfish (*Lophius litulon*) roe on immune response in immunosuppressed mice. Front. Nutr..

[42] Zhang G., Xiao P., Yuan M., Li Y., Xu Y., Li H., Sun J., Sun B. (2023). Roles of sulfur-containing compounds in fermented beverages with 2-furfurylthiol as a case example. Front. Nutr..

[43] Durand E., Laguerre M., Bourlieu-Lacanal C., Lecomte J., Villeneuve P. (2025). Navigating the complexity of lipid oxidation and antioxidation: A review of evaluation methods and emerging approaches. Prog. Lipid Res..

